# Temporal Variability in Diagnosis Code Distributions Across Extraction Time Points in a Multicenter Integrated EHR Database: A Snapshot Comparison Study

**DOI:** 10.1007/s10916-026-02447-5

**Published:** 2026-08-05

**Authors:** Kyunghee Lee, Chihiro Kumagai, Masato Komuro, Kengo Miyo, Hiroyuki Hoshimoto

**Affiliations:** 1 Japan Institute for Health Security, 1-12-1 Toyama, Shinjuku-ku, Tokyo, 162-8655 Japan; 2 Japan Health Research Promotion Bureau, Tokyo, Japan; 3 Medical Artificial Intelligence and Digital Twin in Healthcare, Graduate School of Medicine, The University of Tokyo, Tokyo, Japan

**Keywords:** Electronic health records, Data quality, ICD-10, Multicenter study, Temporal variability, Reproducibility

## Abstract

EHR data are widely used in clinical research; however, their temporal stability remains poorly understood. This study aimed to evaluate temporal variability in EHR data arising from differences in extraction time points. We conducted a retrospective, descriptive, observational study using diagnostic data from a multicenter integrated EHR database in Japan. The target period was 2022, and diagnosis records with a start date within this period were extracted at three time points: October 2023, October 2024, and October 2025. Diagnoses were mapped to the three-character level of the International Classification of Diseases, 10th Revision (ICD-10). Changes in diagnosis code distributions were quantified using the Jensen–Shannon distance (JSD). Changes in record counts per ICD-10 code between 2023 and 2025 were also evaluated. Five facilities were included in the analysis. In the integrated dataset, variations in total records and unique patients were minimal (total records: +0.06% in 2024 and + 0.05% in 2025; unique patients: +0.06% in 2024 and + 0.02% in 2025). JSD values for ICD-10 distributions were non-zero across all facilities, indicating that diagnosis code distributions differed across extraction time points even for the same target period. Across all facilities excluding Facility C, JSD was 0.0022 in 2024 and 0.0026 in 2025 relative to the 2023 baseline. In contrast, Facility C exhibited a markedly larger JSD in 2024 (0.0310), which persisted at a similar level in 2025 (0.0317). At the code level, H61 showed the largest absolute change (− 266 records; −19.98%). Overall, acute conditions tended to decrease, whereas chronic conditions increased across extraction time points. EHR data exhibit temporal variability across extraction time points, even for the same target period. These findings highlight the importance of documenting extraction timing and dataset version, and suggest the need for study designs that explicitly account for such variability.

## Introduction

Electronic health record (EHR) data comprise large volumes of medical information accumulated during routine clinical practice and are widely utilized in clinical epidemiology, healthcare quality assessment, and machine learning applications. In recent years, distributed analytical approaches such as federated learning, which enable the integration and analysis of EHR data across multiple healthcare institutions, have gained increasing attention, highlighting the growing importance of multicenter data research [1, 2]. In such research contexts, ensuring the reproducibility of findings requires a clear understanding of the temporal stability of the underlying EHR data [3, 4].

Previous studies investigating temporal variability in healthcare data have demonstrated that the distribution of EHR data can change over time due to factors such as system updates and revisions to clinical guidelines. For example, a study using laboratory data from a clinical data warehouse in France reported both gradual trends and abrupt changes in many test items, primarily attributed to updates in measurement instruments and software [5]. Similarly, research based on primary care and hospital data in the United Kingdom showed that revisions to clinical coding guidelines led to substantial changes in the frequency of cardiovascular disease codes [4]. However, these studies have focused on distributional changes between data recorded at different time periods within the same database, and it remains unclear whether data recorded for the same target period may vary depending on the timing of data extraction. Studies specifically examining variability arising from differences in extraction timing itself—rather than from changes in clinical practice or coding conventions—remain limited, despite its potential relevance to the reproducibility of multicenter EHR research.

A prior study using a Canadian primary care database reported data integrity issues associated with transitions between EHR systems, including difficulties in linking records across old and new systems due to the absence of common patient identifiers, incorrect overwriting of historical procedure dates, and anomalous reductions in prescription records [6]. These findings suggest that EHR data may be modified even after initial recording due to system updates and data migration processes, raising the possibility that data corresponding to the same target period may differ depending on the extraction time point. Consequently, in study designs that rely on extracting EHR data at different time points for analysis, variations in data composition across extraction times may affect the reproducibility of research findings.

To elucidate such variability associated with extraction timing, it is essential to compare multiple snapshots of data in which all records for the same target period are preserved at different time points. In this context, the 6NC-EHRs database provides a valuable research infrastructure. The 6NC-EHRs is a multicenter integrated database that periodically collects and accumulates EHR data from eight hospitals affiliated with six National Centers for Advanced and Specialized Medicine in Japan, encompassing data from multiple EHR vendors [7]. Because all eligible data are fully extracted at each time point, the database retains complete snapshots of the same target period across multiple extraction times, enabling direct comparison of data variability attributable to extraction timing. The present study makes three specific contributions: (1) it provides quantitative evidence of extraction time point-dependent variability in diagnosis code distributions within a multicenter EHR database; (2) it examines this phenomenon across multiple facilities and EHR vendors; and (3) it identifies patterns of variability that have practical implications for the documentation and governance of EHR datasets used in research. The objective of this study is to quantitatively evaluate temporal variability in EHR data arising from differences in extraction timing and to clarify the temporal stability of EHR data for research use.

## Methods

### Study Design and Data Source

This study was a retrospective, descriptive, observational study designed to evaluate the extent to which EHR data recorded during the same target period vary depending on the timing of data extraction. The data source was the Integrated Database linked to Electronic Health Record of National Centers (6NC-EHRs), which integrates EHR data from six national centers in Japan, managed by the Japan Health Research Promotion Bureau (JH) [8]. The 6NC-EHRs is a multicenter integrated research database that periodically collects and consolidates EHR data from eight hospitals affiliated with six National Centers for Advanced and Specialized Medicine in Japan. Data are extracted in full from the SS-MIX2 standardized storage system via the Multi-purpose Clinical Data Registry System (MCDRS) [9], which is based on the HL7 standard [10, 11]. Because all eligible data are fully extracted at each time point, multiple snapshots are preserved through continuous full-data extraction.

### Study Data

The study data consisted of diagnostic data routinely collected in the 6NC-EHRs database. In addition to diagnosis data, the database includes patient demographics, outpatient and inpatient information, discharge data, prescription orders, injection records, and laboratory test results, structured in accordance with the SS-MIX2 format. Diagnosis data were selected because they are widely used as criteria for cohort definition in database research. Three extraction time points were analyzed: October 2025 (the most recent available at the time of study initiation), October 2024, and October 2023. The target period was defined as January 1 to December 31, 2022, which was common across all three extraction time points. Diagnosis records with a recorded diagnosis start date within this period were included in the analysis. The observational unit for all analyses was the individual diagnosis record.

### Variables

The variables analyzed included the local diagnosis code, diagnosis start date, and facility identifier for each diagnosis record. Local diagnosis codes are identifiers used within each institution’s EHR system and may correspond to MEDIS [12] standardized disease management codes, claims codes, or institution-specific codes, depending on the facility. In this study, local diagnosis codes were mapped to the three-character level of the International Classification of Diseases, 10th Revision (ICD-10, 2013 version) [13] using the MEDIS ICD-10–compatible standard disease master and dental disease master [12]. The mapping process used the type of local code (e.g., disease management code or claims code) as the key and referenced the latest available versions at the time of study initiation (ICD-10 standard disease master Ver. 5.17 and dental disease master Ver. 3.17) [12]. For facilities using institution-specific local codes, these codes were first converted to standardized disease management codes using facility-specific mapping tables, followed by assignment of ICD-10 codes using the same master. Records without a corresponding ICD-10 code were classified as “unassigned.” The diagnosis start date was defined according to the 6NC-EHRs specification, based on either the diagnosis date (PRB-7) or the problem start date (PRB-16) in the SS-MIX2 diagnosis data. Specifically, PRB-7 was used when a diagnosis date was available, whereas PRB-16 was used when the diagnosis date was absent. Although facility identifiers were used to distinguish the source institution of each record, they were anonymized in the manuscript to avoid unnecessary disclosure of institution-specific operational details.

### Selection of Study Sites

To ensure consistency in inter-facility comparisons, only facilities with available data for all three extraction time points were included in the analysis. Facilities lacking data at any of the extraction time points were excluded. In addition, for facilities using institution-specific local codes, only those with available mapping tables at the start of the study were included.

### Statistical Analysis

#### Descriptive Statistics

For each facility and each extraction time point, the following metrics were calculated: total number of records, number of unique patients, number of unique ICD-10 codes, number of records without ICD-10 assignment, and the proportion of records without ICD-10 assignment.

#### Evaluation of Temporal Variability in ICD-10 Distributions

To quantify changes in diagnosis code distributions across extraction time points, the Jensen–Shannon distance (JSD) [3, 4], an information-theoretic measure, was used. JSD ranges from 0 to 1, where 0 indicates identical distributions, and 1 indicates complete divergence [3, 4]. The measure is relatively insensitive to sample size and is suitable for comparing large-scale datasets [3, 4]. In this study, the 2023 extraction was used as the reference, and JSD was calculated based on the proportional distribution of ICD-10 codes (three-character level) for each subsequent extraction year. Records without a valid ICD-10 mapping (“unassigned”) were treated as a separate category in the proportional distributions used for the JSD calculations. Temporal changes were visualized using line graphs. Because all analyses were based on complete extraction snapshots rather than sampled data, JSD was used as a descriptive measure of distributional differences rather than as an inferential statistical measure.

#### Evaluation of Changes by ICD-10 Code

To assess code-level changes, the 2023 and 2025 extraction datasets were compared. For each ICD-10 code, the difference in the number of records and the percentage change was calculated. Codes were ranked by the absolute value of the difference, and the top 10 codes with the largest changes were identified. All analyses were conducted using R version 4.4.3 [14]. This study was approved by the ethics committee of the National Center of Neurology and Psychiatry (approval number: B2020-139).

## Results

A total of five facilities (Facility A-E) had available data for the target period across all three extraction time points (October 2023, October 2024, and October 2025) and were included in the analysis. Facilities F and G were excluded because data were unavailable for all three time points. In addition, Facility H was excluded because the required mapping table was not available at the time of study initiation.

### Descriptive Statistics

An overview of the data across the three extraction time points is presented in Table 1.

**Table 1:** Overview of data by facility and extraction year

Facility (vendor)	Extraction year	Total records		Unique patients		Unique ICD-10 codes (three-character)^¶^		Unassigned ICD-10 records^¶^		Proportion of unassigned ICD-10 records^¶^ (%)
		*n*	Difference from baseline	*n*	Difference from baseline	*n*	Difference from baseline	*n*	Difference from baseline	
ALL	2023	775,058	ー	103,833	ー	1,332	ー	3,682	ー	0.475
	2024	775,528	470 (+ 0.06%)	103,891	58 (+ 0.06%)	1,334	2 (+ 0.15%)	3,673	-9 (-0.24%)	0.474
	2025	775,475	417 (+ 0.05%)	103,853	20 (+ 0.02%)	1,334	2 (+ 0.15%)	3,672	-10 (-0.27%)	0.474
Facility A (Vendor A)	2023	176,787	ー	14,991	ー	914	ー	286	ー	0.162
	2024	178,924	2137 (+ 1.21%)	15,124	133 (+ 0.89%)	915	1 (+ 0.11%)	288	2 (+ 0.70%)	0.161
	2025	179,237	2450 (+ 1.39%)	15,125	134 (+ 0.89%)	915	1 (+ 0.11%)	288	2 (+ 0.70%)	0.161
Facility B (Vendor B)	2023	221,878	ー	37,955	ー	1,233	ー	384	ー	0.173
	2024	221,642	-236 (-0.11%)	37,929	-26 (-0.07%)	1,233	0 (+ 0.00%)	385	1 (+ 0.26%)	0.174
	2025	221,443	-435 (-0.20%)	37,897	-58 (-0.15%)	1,233	0 (+ 0.00%)	384	0 (+ 0.00%)	0.173
Facility C (Vendor B)	2023	45,657	ー	11,707	ー	858	ー	175	ー	0.383
	2024	44,368	-1289 (-2.82%)	11,669	-38 (-0.32%)	857	-1 (-0.12%)	162	-13 (-7.43%)	0.365
	2025	44,263	-1394 (-3.05%)	11,660	-47 (-0.40%)	856	-2 (-0.23%)	161	-14 (-8.00%)	0.364
Facility D (Vendor A)	2023	159,062	ー	15,674	ー	808	ー	2,783	ー	1.75
	2024	159,017	-45 (-0.03%)	15,668	-6 (-0.04%)	810	2 (+ 0.25%)	2,783	0 (+ 0.00%)	1.75
	2025	158,999	-63 (-0.04%)	15,664	-10 (-0.06%)	810	2 (+ 0.25%)	2,783	0 (+ 0.00%)	1.75
Facility E (Vendor B)	2023	171,674	ー	23,506	ー	944	ー	54	ー	0.031
	2024	171,577	-97 (-0.06%)	23,501	-5 (-0.02%)	945	1 (+ 0.11%)	55	1 (+ 1.85%)	0.032
	2025	171,533	-141 (-0.08%)	23,507	1 (+ 0.00%)	945	1 (+ 0.11%)	56	2 (+ 3.70%)	0.033

¶ ICD-10 codes are presented at the three-character level

In the integrated dataset across all five facilities, the total number of records showed minimal variation: 775,058 in 2023, 775,528 in 2024 (+ 470; +0.06%), and 775,475 in 2025 (+ 417; +0.05%). Similarly, the number of unique patients was 103,833 in 2023, 103,891 in 2024 (+ 58; +0.06%), and 103,853 in 2025 (+ 20; +0.02%).

At the facility level, variability in the total number of records and the number of unique patients differed across sites. At Facility A, the number of unique patients increased from 14,991 to 15,125 (+ 134; +0.89%). In contrast, at Facility C, the total number of records decreased from 45,657 in 2023 to 44,263 in 2025 (− 1,394; −3.05%), representing a larger change than at other facilities (|0.03–1.39|%). The number of unique ICD-10 codes at the three-character level was 1,332 in 2023 and 1,334 in both 2024 and 2025 in the overall dataset, indicating slight variation across extraction time points. At the facility level, no change was observed at Facility B (0%), whereas the magnitude of change was similar across the remaining facilities (|0.11–0.25|%). The number of records without an ICD-10 assignment also varied across facilities. At Facility C, the number decreased from 175 in 2023 to 161 in 2025 (− 14; −8.00%), representing a larger relative change compared with other facilities (|0–3.70|%). No clear differences in patterns were observed across EHR vendors for any of the evaluated metrics, including total records, unique patients, unique ICD-10 codes, number of unassigned records, and proportion of unassigned records.

### Temporal Variability in ICD-10 Distributions

**Fig. 1:**
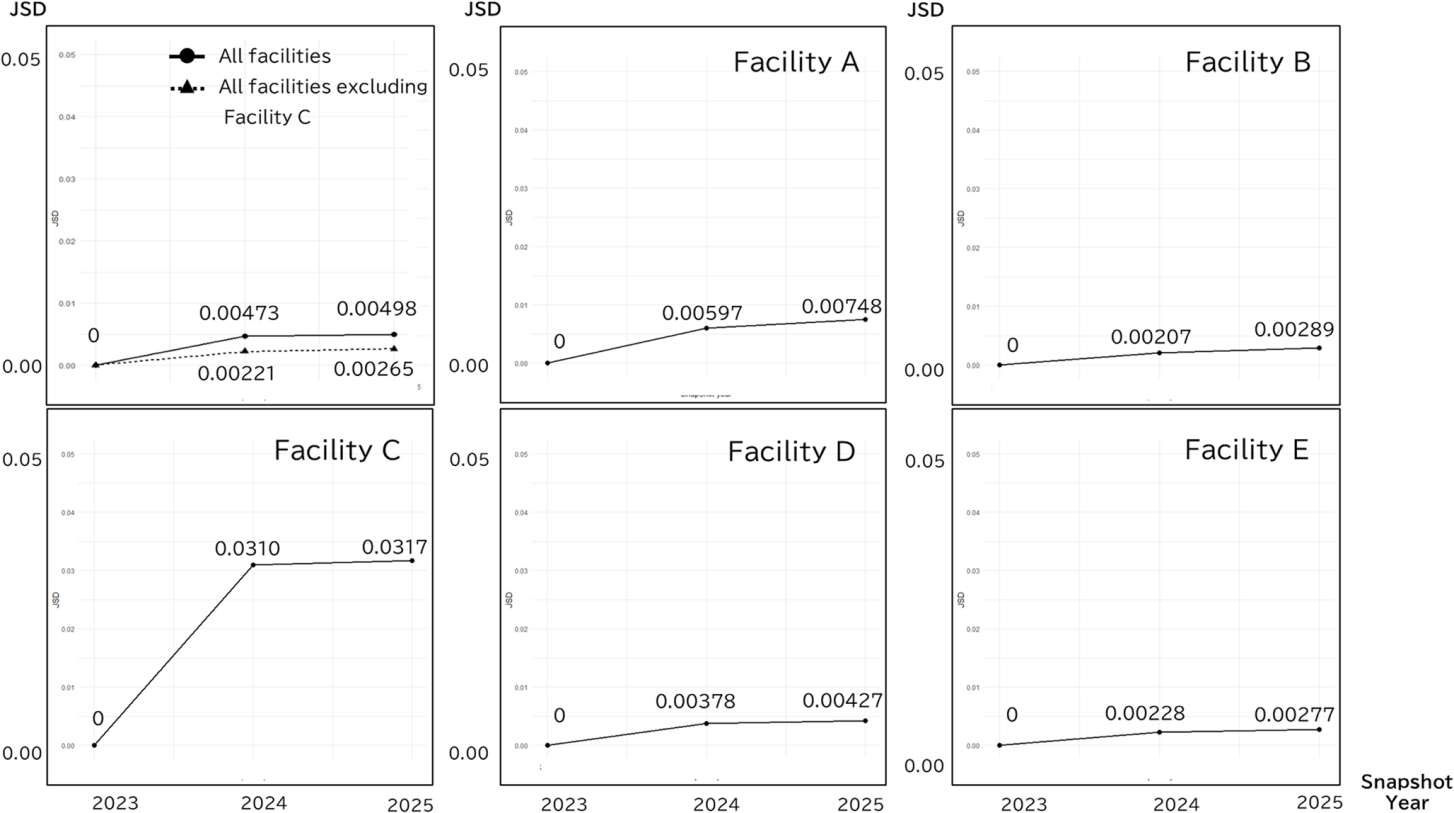
Jensen–Shannon distance (JSD) of ICD-10 distributions from the baseline year (2023). JSD was calculated between the October 2023 extraction and each subsequent extraction time point (October 2024 and October 2025), based on the proportional distribution of ICD-10 codes at the three-character level. The upper-left panel displays results for all five facilities combined (solid line with filled circles) and for all facilities excluding facility **C** (dashed line with filled triangles). The remaining panels show facility-level results. The y-axis scale is consistent across all panels (0 to 0.05)

Figure 1 presents the temporal trends in JSD for the proportional distribution of ICD-10 codes (three-character level), using the 2023 extraction as the reference. Across all facilities, the JSD values for the 2024 and 2025 extractions were non-zero, indicating differences in diagnosis code distributions across extraction time points despite the same target period. The magnitude of these differences varied across facilities. While Facilities A, B, D, and E exhibited only slight increases in JSD across extraction time points, with JSD values remaining at or below 0.00748, Facility C showed a marked increase to 0.0310 in 2024, which persisted in 2025 (0.0317).

### Changes by ICD-10 Code

Table 2 presents the top 10 ICD-10 codes (three-character level) with the largest absolute changes in record counts between the 2023 and 2025 extractions. The largest change was observed for H61 (other disorders of the external ear), which decreased from 1,331 records in 2023 to 1,065 in 2025, representing a 266-record reduction (− 19.98%). This was followed by E11 (type 2 diabetes mellitus), which increased by 191 records (+ 1.73%). The third-largest change was observed for U07 (emergency use of U07, primarily representing COVID-19), which decreased by 108 records (− 0.33%). Among the codes with the largest changes, several were related to chronic conditions, including E11 and E14 (unspecified diabetes mellitus), I10 (essential [primary] hypertension), K59 (other functional intestinal disorders), and G30 (Alzheimer’s disease).

**Table 2:** Top 10 ICD-10 codes with the largest absolute changes in record counts between 2023 and 2025

Rank	ICD10^¶^		Records in 2023 (proportion, %)^§§^	Records in 2025 (proportion, %)^§§^	Difference from baseline
1	H61	Other disorders of external ear	1,331(0.17)	1,065(0.14)	-266 (-19.98%)
2	E11	Type 2 diabetes mellitus	11,022(1.42)	11,213(1.45)	191 (+ 1.73%)
3	U07	Emergency use of U07^¶¶^	32,289(4.17)	32,181(4.15)	-108 (-0.33%)
4	I10	Essential (primary) hypertension	10,357(1.34)	10,463(1.35)	106 (+ 1.02%)
5	K59	Other functional intestinal disorders^§^	8,504(1.10)	8,588(1.11)	84 (+ 0.99%)
6	J02	Acute pharyngitis	2,563(0.33)	2,480(0.32)	-83 (-3.24%)
7	K21	Gastro-esophageal reflux disease	6,241(0.81)	6,313(0.81)	72 (+ 1.15%)
8	E14	Unspecified diabetes mellitus	16,500(2.13)	16,431(2.12)	-69 (-0.42%)
9	G30	Alzheimer disease	4,124(0.53)	4,185(0.54)	61 (+ 1.48%)
10	K05	Gingivitis and periodontal diseases	4,914(0.63)	4,860(0.63)	-54 (-1.10%)

¶ ICD-10 codes are presented at the three-character level

¶¶ Primarily represents COVID-19

§ Primarily represents constipation

§§ Proportion relative to the total number of records in the respective year

## Discussion

This study demonstrated that, within a multicenter integrated EHR database, diagnosis data recorded for the same target period exhibit measurable variability depending on the timing of data extraction. Across all facilities, the JSD values for the 2024 and 2025 extractions were non-zero when compared with the 2023 extraction. This finding indicates that, even for data from the same target period, diagnosis code distributions do not fully coincide across different extraction time points. Nevertheless, the observed variability remained low across most facilities throughout the study period.

In this study, JSD changed between the 2023 and 2024 extractions across all facilities and subsequently showed gradual trends through 2025. Because this study was based on repeated cross-sectional snapshots rather than longitudinal tracking of individual patients, the underlying mechanisms could not be directly verified. Therefore, the following interpretations should be regarded as hypotheses. First, a revision of laboratory test item definitions was implemented in May 2024 in the 6NC-EHRs database via MCDRS [9], including the addition of test items and changes to the data fields used to capture test execution dates; these modifications may have affected data extraction across all facilities, potentially leading to changes in record counts. Second, it has been widely noted that EHR data are inherently dynamic, and clinical records are subject to retrospective modifications regardless of the specific extraction system [15, 16]. Systematic reviews highlight that delays in updating problem lists—such as the delayed deletion of past conditions or the delayed entry of newly diagnosed issues—are universal challenges in EHR data quality [16]. Consistent with this, previous studies from England and Wales have reported that, at the time of extraction from EHR systems, recent clinical events may not yet be fully coded and are subsequently retrospectively updated at different rates across facilities [17]. Our findings may be partly consistent with these previous studies. In Japan, for reimbursement purposes, provisional diagnoses may be temporarily recorded at the time of testing before confirmation, and subsequently modified or removed following diagnostic confirmation—for example, by removing qualifiers such as “suspected,” replacing the diagnosis, or deleting the record entirely. Under such practices, initially recorded diagnoses may be revised or removed over time, leading to a reduction in their frequency in later data extractions. In this study, decreases were observed in acute conditions such as COVID-19 and acute pharyngitis, which were prevalent during the target period (2022). This pattern may reflect the influence of provisional diagnoses recorded for testing purposes that were later revised or removed. The largest decrease was observed for H61 (other disorders of the external ear), which includes conditions such as chondritis of the external ear, cerumen impaction, and external auditory canal stenosis. These diagnoses may also have been temporarily recorded for diagnostic purposes; however, this study could not further elucidate the underlying mechanisms. It should be noted that the relative contributions of these two factors could not be separately assessed based on the available data.

In contrast, many of the codes that increased from the baseline year were related to chronic conditions, including diabetes mellitus and hypertension. One possible explanation is the nature of the participating institutions in the 6NC-EHRs database, which are advanced, specialized medical centers. Patients treated at such institutions are often referred from other healthcare providers, and chronic comorbidities may be newly documented at the time of consultation. In these cases, the recorded diagnoses typically represent pre-existing conditions rather than newly incident diseases, and the diagnosis start date may be retrospectively assigned to an earlier onset. Consequently, diagnoses entered at later visits may be retrospectively reflected in data for the target period (2022). The increasing prevalence of multimorbidity may also contribute to this pattern. Previous studies have reported that 64.9% of individuals aged 65–84 years and 81.5% of those aged ≥ 85 years have multiple chronic conditions [18], and in Japan, approximately 65% of individuals aged ≥ 75 years have three or more chronic diseases [19]. These factors may underlie the observed increase in chronic disease codes. In addition to the post-extraction record modifications reported in prior studies [17], our findings suggest that retrospective documentation of chronic conditions at subsequent encounters may result in the addition of diagnosis records for prior target periods. Whether the observed increase in chronic condition codes was associated with the age distribution of patients at participating facilities could not be verified, as patient-level demographic data were not analyzed in this study.

When examining inter-facility differences, distinct patterns of variation were observed in both total record counts and JSD, with Facility C showing the greatest variability and a pattern distinct from those of the other facilities. At Facility C, a major EHR system update was implemented in January 2024, which may have contributed to the changes observed between the October 2023 and October 2024 extractions. Previous studies from Canada [6] have similarly reported structural changes in data associated with EHR system transitions, and our findings are consistent with these observations. At Facility B, a software update was also implemented in December 2023; however, because it was not a major system change, the impact on data variability appears to have been limited. No clear differences in variability patterns were observed across EHR vendors. At Facility A, increases in total records and unique patients were observed; however, the underlying causes could not be identified based on the available data. Further investigation, including review of facility-level system logs and data management records, would be warranted.

Overall, these findings indicate that, in studies using EHR data, not only the target period but also the timing of data extraction itself can influence study results. Because EHR data are dynamic and subject to modification and augmentation after initial recording, differences in extraction timing may alter the dataset’s composition, potentially affecting the reproducibility and comparability of findings [15, 20]. Furthermore, in multicenter studies, differences in system update timing and data revision processes across institutions may introduce technical variability that does not reflect true differences in disease patterns [3, 17]. Therefore, wherever feasible, it is recommended that researchers and data administrators record not only the study period and extraction conditions but also the extraction date and dataset version used for analysis [20, 21].

This study has several limitations. First, the analysis was limited to diagnosis data; thus, it remains unclear whether similar variability exists in other clinical domains, such as laboratory results or medication records. Second, because only three extraction time points were examined, longer-term variability patterns could not be assessed. Furthermore, because all extractions were performed in October, the effects of time since the target period could not be distinguished from potential extraction-month effects. Therefore, the observed temporal trajectory of JSD may not be generalizable to different extraction schedules or calendar months. Third, although temporal correspondence was observed between facility-level system updates and data variability, we could not directly link these factors to specific data output specifications, precluding definitive causal conclusions. Fourth, the study population comprised advanced specialized medical centers, so the findings may not be fully generalizable to other healthcare settings. In addition, three eligible facilities were excluded because data for one or more extraction time points or the required mapping tables were unavailable. As these facilities were not analyzed, we could not assess whether they differed systematically from the included facilities, and therefore the observed variability patterns may not fully represent all facilities participating in the 6NC-EHRs database. Fifth, and importantly, our analysis relied on repeated cross-sectional snapshots of extracted data rather than tracking individual patient trajectories. Consequently, our mechanistic explanations for the observed distributional changes—namely, the retrospective addition of chronic conditions and the delayed deletion of provisional acute diagnoses—remain hypotheses. Future research linking patient-level longitudinal records across multiple extractions is required to directly investigate how individual clinical entries are modified post-recording and to validate these mechanisms.

## Conclusion

In this study, a comparison of temporal snapshots from a multicenter integrated EHR database demonstrated that diagnosis data recorded for the same target period exhibit temporal variability depending on the timing of data extraction. The magnitude of this variability may be influenced by facility-specific system updates. These findings highlight the importance of explicitly documenting extraction timing and dataset version in EHR-based research and suggest the need to account for this variability in study design and interpretation.

## Data Availability

The data used in this study are not publicly available due to regulations of the 6NC-EHRs data utilization program managed by JH. Access to the data is restricted to researchers approved under the 6NC-EHRs data utilization program and is granted upon completion of the appropriate application and approval procedures.
